# Early Pediatric Benefit of Lutein for Maturing Eyes and Brain—An Overview

**DOI:** 10.3390/nu13093239

**Published:** 2021-09-17

**Authors:** Diego Gazzolo, Simonetta Picone, Alberto Gaiero, Massimo Bellettato, Gerardo Montrone, Francesco Riccobene, Gianluca Lista, Guido Pellegrini

**Affiliations:** 1 Neonatal Intensive Care Unit, Department of Pediatrics, University G. d’Annunzio, 65100 Chieti, Italy; 2 Department of Pediatrics, Maastricht University, 6200 MD Maastricht, The Netherlands; 3 Neonatal Intensive Care Unit, Policlinico Casilino, 00169 Rome, Italy; simpico@libero.it; 4 Pediatric and Neonatology Unit, asl2 Ospedale San Paolo Savona, 17100 Savona, Italy; a.gaiero@asl2.liguria.it; 5 Department of Women and Child’s Health, San Bortolo Hospital, 36100 Vicenza, Italy; massimo.bellettato@aulss8.veneto.it; 6 S.S.V.D “NIDO E STEN” Ospedali Riuniti Foggia, 71122 Foggia, Italy; montrone.gerardo@gmail.com; 7 UOC Nido d’Osservazione–San Camillo Forlanini Hospital, 00152 Rome, Italy; riccobenefrancesco@gmail.com; 8 Neonatal Intensive Care Unit, Department of Pediatrics, Ospedale dei Bambini V. Buzzi, ASST-FBF-Sacco, 20154 Milan, Italy; gianluca.lista@asst-fbf-sacco.it; 9 Department of Pediatrics and Neonatology, Presidio Ospedaliero “Città di Sesto San Giovanni, Sesto san Giovanni, 20099 Milan, Italy; guido.pellegrini@asst-nordmilano.it

**Keywords:** lutein, zeaxanthin, macular pigment, oxidative stress, blue-light, eye development, brain development, visual function, cognitive function, carotenoids, nutrition

## Abstract

Lutein is a dietary carotenoid preferentially accumulated in the eye and the brain in early life and throughout the life span. Lutein accumulation in areas of high metabolism and oxidative stress such as the eye and the brain suggest a unique role of this ingredient during the development and maturation of these organs of common embryological origin. Lutein is naturally provided to the developing baby via the cord blood, breast milk and then infant diet. The presence of this carotenoid depends on fruit and vegetable intakes and its bioavailability is higher in breastmilk. This paper aims to review the anatomical development of the eye and the brain, explore the presence and selective deposition of lutein in these organs during pregnancy and infancy and, based on its functional characteristics, present the latest available research on the beneficial role of lutein in the pediatric population. The potential effects of lutein in ameliorating conditions associated with increase oxidative stress such as in prematurity will be also addressed. Since consumption of lutein rich foods falls short of government guidelines and in most region of the world infant formulas lack this bioactive, dietary recommendations for pregnant and breastfeeding women and their child can help to bridge the gap.

## 1. Introduction

Nutrition during pregnancy is important for maternal health, pregnancy outcomes, fetus development child health, and potentially later in life [1].

Perinatal development and infancy are life stages characterized by multiorgan development. Poor maternal/fetal/neonatal nutrition can influence developmental “programming” and organ growth resulting in short-term and longer-term effects linked particularly to the increased risk of noncommunicable diseases [2].

The WHO recommends nutrition education and counselling during pregnancy about healthy diet which contains macronutrients, vitamins and minerals, obtained through the consumption of a variety of foods, including green and orange vegetables, meat, fish, beans, nuts, pasteurized dairy products and fruits [3].

Development, infancy, and early childhood are a particular critical window for eye and brain development. Preterm birth contributes to neonatal morbidity, physical and neurodevelopmental disabilities.

Traditional evidence generated from clinical studies and the related guidelines mainly address the prevention of deficiencies of essential macro and micro-nutrients. Researchers have recently started to explore the role of non-essential bio-actives on the promotion of optimal health.

One of the bio-actives considered important for eye and brain development is lutein.

### 1.1. Retina and Brain Development

The retina and the brain share the same embryological origin from the neural tube formed from the neural plate around the 4th week of gestation (WG) [4]. In particular, in the embryonic period the formation of three brain vesicles constitutes the primary regions of the brain as follows: the prosencephalon (future forebrain), mesencephalon (future midbrain) and the rhombencephalon (future hindbrain).

**Figure 1:**
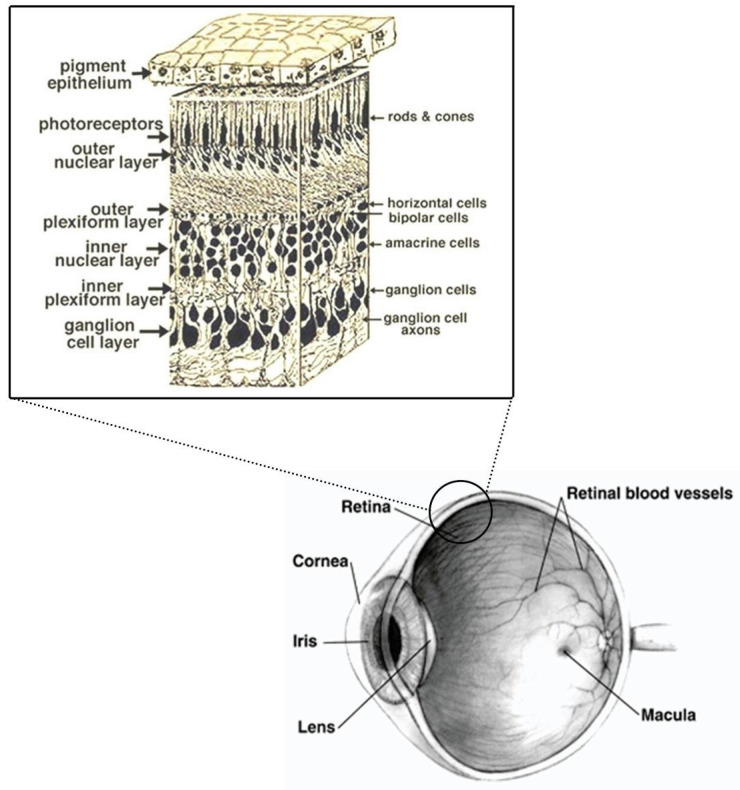
Eye and retina section scheme adapted from Webvision, (http://webvision.med.utah.edu/) and NIH National Eye Institute.

**Figure 2:**
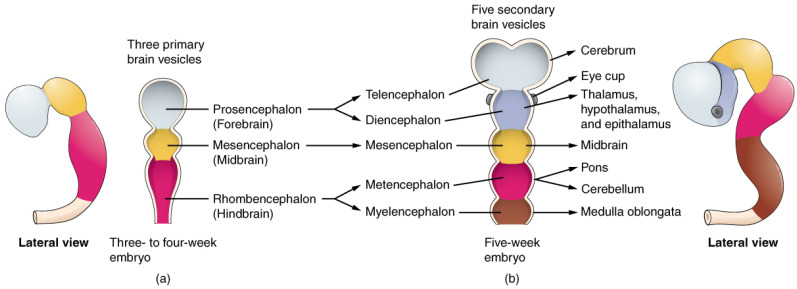
Primary and Secondary Vesicle Stages of Development The embryonic brain develops complexity through enlargements of the neural tube called vesicles; (**a**) The primary vesicle stage has three regions, and (**b**) the secondary vesicle stage has five regions. Credit: OpenStax.

The prosencephalon develops further into the telencephalon, the diencephalon, and a pair of optic vesicles. These structures give rise to the cerebral hemispheres, the thalamus/hypothalamus and the optic nerve, retina, and iris. The rudimentary structures of the eye are distinguishable by −5 WG (Figure 1 and Figure 2).

The visual system develops rapidly during the first years of life. Retinal development begins centrally before extending peripherally. The ganglion cell layer, inner plexiform layer, inner and outer nuclear layer can be observed in the center of the fetus retina at 20–22 WG. At 25 WG, the foveal pit begins to form and then it continues to deepen during the subsequent weeks up to the end of pregnancy concomitantly with the displacement of the ganglion cell layer, inner plexiform layer, inner nuclear layer to the periphery [5]. The retina matures in a centripetal direction, the peripheral retina is fully developed, and the fovea is immature at birth and matures over several years. A steep increase in cone density in the fovea with displacement of rod photoreceptors is observed from 22 WG (18,000 cells/mm^2^) to 4 years of age (108,000 cells/mm^2^). Maturation of foveal cones and elongation of their outer segments occur postnatally and is responsible, together with the maturation/plasticity of the visual circuits, for response to the visual stimulus and for increase of visual function in the postnatal period.

The retinal vasculature starts to develop at approximately 16 WG and continues up to the end of pregnancy with the vascularization of the peripheral retina. The formation of these vessels is regulated by physiological hypoxia. The eye reaches approximately half the adult size at six fetal months and two thirds of the adult size by term. After birth, the axial length continue to increase at a progressive slow pace up to the age of 14–15 years [6,7].

The developing human brain also experiences a period of rapid growth, beginning approximately mid-pregnancy and continuing through the first few years after birth, leading to a large increase in brain volume including a large expansion in lipid content. Brain development is composed of different stages overlapping in time and characterized by proliferation of undifferentiated brain cells, migration of the cells to predefined locations, contextual differentiation in specific cell types and aggregation into distinct regions, formation of intra/inter region connections, remodeling and stabilization of these connections [8].

At six months, the cerebral cortex covers most of the other brain structures and starts to separate into lobes, the fetus brain waves can be detected by the seventh month of pregnancy. Concerning the visual cortex, the ocular dominance columns which then develop into the directional columns, begin to form during the last 8–10 WG. Further development occurs after birth in response to the visual stimulation and experience. Further reorganization of the visual system presumably occurs also in association with the development of the ability to learn, to read words and recognize them occurring after 5 years of age [8,9].

Most of the brain rapid growth is postnatal. The cut-off point of weight accumulation is between 18 postnatal months and 2 years for whole brain and forebrain while the phase of rapid myelination ends at about the age of four years [10]. The pruning of excess connection continues for years. 

The eye and the brain are complex and unique organs that develop and change throughout life. In the early years—ranging from conception throughout the first years of life—both go through the biggest transformation. The complexity and the length of the eye and the brain development make them particularly vulnerable to the effects of environment insults so it is important to ensure the best environmental/nutritional conditions to promote human eye and brain growth and development in this period.

### 1.2. Oxidative Stress in the Term and Preterm Newborn

Birth exposes the infant to increased risk for oxidative insult due to an oxygen concentration gradient between intra (oxygen saturation of fetal blood of about 65%) extrauterine life (90% saturation at 5 min of age) and the presence of non-protein bound iron (particularly in preterm infants) [11,12,13]. Therapeutic strategies performed in the delivery room or in neonatal intensive care unit (NICU) can also increase free radical’s production. The negative effects of oxidative and nitrogen stress may also start early during pregnancy. The retina and the brain are particularly susceptible to oxidative stress (OS). The high metabolic activity, oxygen consumption and presence of polyunsaturated fatty acids, constituent lipids of neuronal membranes, favor the generation of free radicals and the propagation of the insult. The immaturity of the antioxidant (AO) endogenous system in terms of concentration of AO enzymes and their activity also contributes to the OS propensity [14,15]. Perinatal hypoxic/ischemic events may lead to increased organ/tissues damage and reperfusion maneuvers can exacerbate the damage further [11,15]. 

Premature birth increases the risk for OS. Preterm babies are more likely to die and to have short and long-term morbidities. Common complications includes high rates of respiratory distress syndrome, bronchopulmonary dysplasia (BPD), necrotizing enterocolitis (NEC), sepsis, periventricular leukomalacia, seizures, intraventricular hemorrhage, cerebral palsy, infections, feeding difficulties, hypoxic ischemic encephalopathy, and hearing and visual problems than those born at term [16]. OS and inflammation have been implicated in the pathogenesis of most of these complications [11].

Protecting the newborn infant against perinatal OS is a healthcare priority, and therefore the search for new, safe, and efficacious AO has been a major focus of research during the last decade. Among the diverse approaches to attenuate OS and optimize development, research has explored the role of lutein. Lutein is a dietary compound with well know AO characteristics, decades of investigation in age-related eye diseases and emerging evidence on the beneficial effect in cognitive health and function. 

### 1.3. Light and the Eye

The electromagnetic (EM) energy in the solar spectrum that reaches earth surface is broken down into about 7% ultraviolet (UV) radiation (wavelength 290–400 nm), 44% visible “white” light (VL) (400–700 nm spanning the entire rainbow color range from violet to red), and 49% infrared (IR) radiation (200–2500 nm) [17]. For what concerns UV and infrared radiation, while the sun emits optical radiation over the full wavelength range, the earth’s atmosphere absorbs UVC (100–280 nm), the majority of the UV irradiation between 290 and 320 nm and IRC of wavelengths over 30 μm [17,18]. The penetration of the different wavelengths of the optical radiation into the eye depends on the interaction with the diverse eye structures.

**Figure 3:**
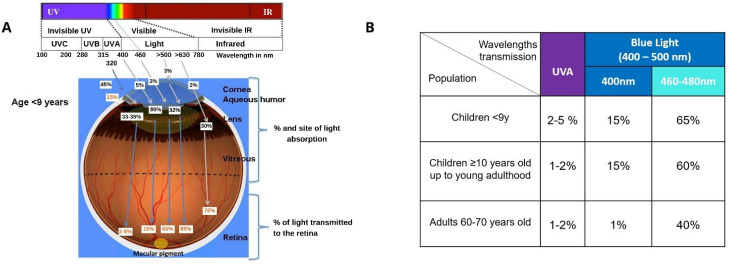
(**A**) Specificity of light interaction with the eye of children below the age of 9 years in agreement with SCENIHR (reproduced from SCENIHR 2012 [18]). (**B**) Transmission of ultraviolet A (UVA) and blue light to the retina of <9 years old, children 10 years old up to young adulthood and the eye of a 60–70 years old adult in agreement with SCENIHR.

The human cornea absorbs over 90% of the wavelengths between 300–320 nm (UVB range), about 30–40% between 320–360 nm (UVA range) and almost all the IR radiation above 800 nm (i.e., IRA, IRB and IRC ranges). UVB and 45–50% of the UVA transmitted by the cornea are then absorbed by the lens and part of the UVA transmitted by the lens is absorbed by the vitreous resulting in only 1–2% of UVA radiation and all the VL wavelengths reaching the retina. This filtering system is less functional in young subjects of school age or younger (approximately less than 9 years old) resulting in a transmission of up to 5% UVA to the retina [18,19]. As depicted in Figure 3, age-related differences in the transmission optic radiation to the retina are also observed for the most energetic wavelengths of VL radiation—blue light (400–500 nm). In infant, children and young adults around 15% of 400 nm and about 60–65% of 460–480 nm wavelengths reach the retina while, due to the progressive yellowing of the lens, only about 1% of blue light at 400 nm and 40% at 460–480 nm reaches the retina of a senior person. A person over the age of 60 has a blue-light filtration rate around twice that of a 20-year-old [20]. Thus, the retina of children is particularly exposed to shorter wavelengths of optical radiation (UVA and blue light) which have the greater potential for generation of reactive oxygen species (ROS) and biological damage. 

Ocular damage from light can occur through either an inflammatory response or a photooxidation reaction often mediated by eye chromophores (rhodopsin, opsin, melanin or A2E found in lipofuscin granules) that absorb light visible range and generate Reactive Oxygen Species (ROS). Phototoxic damage can occur in the retinal pigment epithelium (RPE), the choroid and the photoreceptors [21]. Significantly, the observations of Wing et al. [22] suggest that the greatest rate of increase in lipofuscin formation in the RPE is found during infancy in the first 5–6 years of post-natal life. Moreover, lipofuscin is a biomarker of ageing and its accumulation increases in an age-dependent manner.

Evidence from epidemiological and experimental studies suggest that cumulative exposure to blue light may result in short and long term effects for the eye such as blurred vision, eye strain, eye fatigue, retinal damage, reduced visual performance and age-related eye conditions [23,24]. Aside from the potential damage to the structures of the eye, blue light can impact visual discrimination and range in the outdoors, is associated with glare (viewing conditions in which a person experiences discomfort or is less capable of perceiving details or objects, due to an unfavorable luminance distribution or an extreme contrast), and disruption of circadian rhythm (when people are exposed at night) [20,25,26]. 

The augmented susceptibility of the young population to blue light discussed above is considerably important due to the increased exposure to blue light from LED artificial illumination and digital devices. LED lighting systems increase the imbalance in wavelengths in favor of blue light. An Italian observational, cross-sectional study examining a possible relationship between exposure to videogames/electronic screens and visual issues in healthy children 3 to 10 years of age found that prolonged use of videogames in children can impact the development of their visual pathways. Children who played video games for 30 min or more every day presented more symptom of eyestrain, nervous tics, lower percentage of stereopsis and higher prevalence of refractive errors (mainly in the dominant eye) when compared to children who played video games for less than 30 min per day and not every day. The effects were generally more pronounced with concomitant use of other electronic screens (TV, computer tablets and smartphones) for 3 h or more per day [27]. Data from Kim et al. and, more recently confirmed by the expert committee of the French Agency for Food, Environmental and Occupational Health and Safety, show higher prevalence rates for ocular symptoms (blurring, redness, visual disturbance, secretion, inflammation, lacrimation and dryness) related to eye fatigue and strain in adolescents with greater exposure (>2 h/day) to smartphones [20,28].

## 2. Lutein

### 2.1. Physico-Chemical Characteristics

Lutein [(3R, 3′R, 6′R)-β, ε-carotene-3,3′-diol], commonly found in nature together with its isomer zeaxanthin [(3R, 3′R)-β,β-carotene-3,3′-diol], is a lipophilic pigment member of the xanthophyll family of dietary carotenoids. Lutein has no provitamin A activity and represent one of the six major carotenoids circulating in human blood [29]. Similar to other carotenoids it is a tetra-terpenoid composed of a central carbon linear chain of conjugated double bonds and possess one hydroxylated ionone ring at each end of the polyene chain [30,31]. The conjugated polyene chromophore is the most characteristic feature of carotenoids and, together with the end groups, determines the shape, chemical reactivity and consequent role as antioxidants, light-absorbing properties and related color [31,32]. The specific end groups type in the lutein molecular structure (one β ring conjugated with the polyene chain and one non-conjugated ε ring) result in the presence of 10 conjugated double bonds and characterize the molecule ability to absorb light in the blue-band of the visible spectrum (maximum absorption at 445 nm in methanol and 452 nm in olive oil) defining its yellow color [33,34].

Lutein isomer zeaxanthin presents two β rings in the molecule. The presence of these end groups increase the number of conjugated double bonds to eleven and results in a shift of maximum light absorption to a slightly higher wavelength within the blue light band (maximum absorption at 450 nm in methanol and 463 nm in olive oil) and a more intense yellow-orange color [33,34]. Because of the C=C isomerism, multiple cis/trans configurations are theoretical possible for lutein and zeaxanthin. Furthermore, the hydroxyl groups in the 3 and 3 carbon of the end group generates chiral centers resulting in R and S stereoisomer. Only a few of these isomers are present in nature and in the typical diet lutein and zeaxanthin are predominantly found in the thermodynamically more stable trans-form and exclusively as R stereoisomers [31,32,34].

**Figure 4:**
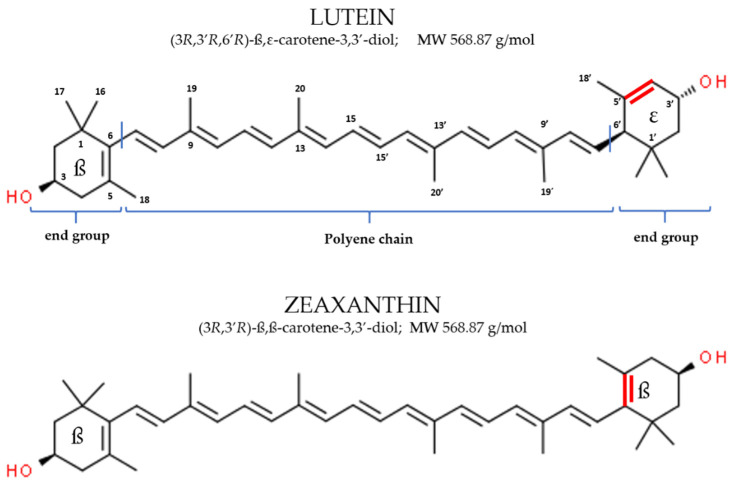
Chemical structure of dietary lutein and zeaxanthin.

**Figure 5:**
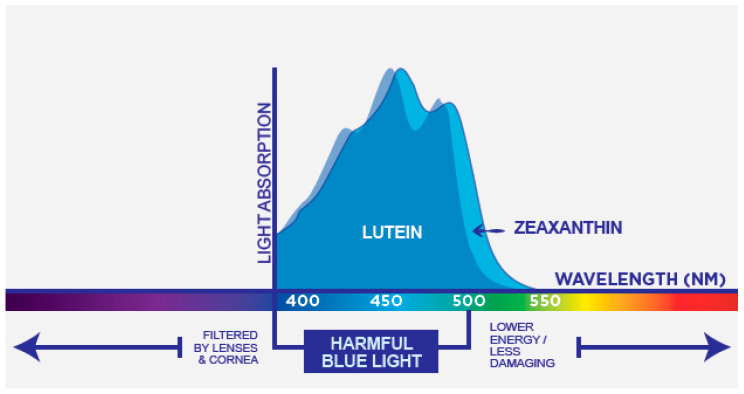
Absorption spectrum of lutein (blue) and zeaxanthin (light blue).

The chemical structure of lutein and zeaxanthin and their absorption spectra is presented in Figure 4 and Figure 5, respectively.

### 2.2. Dietary Sources, Absorption and Bioavailability

Animals do not synthesize carotenoids *de novo*, and so the carotenoids found in the body are either directly obtained from food or partly modified from ingested foods through metabolic reactions [30]. The main dietary sources of carotenoids are usually fruits and vegetables.

Lutein is among the most abundant carotenoids in the human diet [31] often found combined with zeaxanthin. Lutein and zeaxanthin combined represent approximately 20% of the total carotenoids [35,36]. Richest food sources of lutein are all dark green leafy vegetables in particular kale and spinach which contain 40 mg and 12 mg lutein in 100 g serving, respectively [37].

Zeaxanthin is generally found in much lower amounts in vegetables and is the dominant xanthophyll in only a few food products, such as the goji berry and orange pepper. Lutein and zeaxanthin are also present in foods of animal origin such as egg yolk. They can additionally be used as colorants in diverse beverages and are found commercially in dietary supplements. In fruits, xanthophylls with a hydroxyl group such as lutein and zeaxanthin may occur as fatty acid esters. Due to the fact that their concentration in fruit is generally low, compared to vegetables, the human diet is predominantly constituted by lutein and zeaxanthin in the free form, which is also the only form directly absorbed by the body [35,36,37,38].

Lutein consumed from dietary sources exceeds the amounts of zeaxanthin being consumed typically at a dietary ratio of 5:1. This lutein:zeaxanthin ratio has been extensively explored in clinical trials. Studies conducted in patients suffering from age-related macular degeneration (AMD) have shown the benefits of supplementation with 10 mg lutein in combination with 2 mg zeaxanthin or with other amounts in improving visual function and delaying the progression of AMD to more advanced disease stages [39,40,41,42]. Additional studies conducted in a healthy young or more senior adult population have shown that 10 mg lutein + 2 mg zeaxanthin supplementation increases MPOD and provide benefits on visual and cognitive health and function [43,44,45,46,47,48,49]. Although possible (10 mg of lutein are found in 80 g of spinach), getting these nutrients in the adequate amount through the diet alone can be difficult on a regular daily basis especially in childhood.

Estimates of lutein and zeaxanthin intake from the US National Health and Examination Survey (NHANES) by individuals 2 years old and older consuming the 3 to 5 serving of diverse fruit and vegetable recommended by the dietary guidelines for Americans indicate that the mean consumption of lutein and zeaxanthin is 3.83 mg/day reaching 7.29 mg/day in the 90th percentile of intake [50]. Toddlers, in this well-nourished population, ingest 3.2 mg of lutein and zeaxanthin per day (90th percentile). However, intake data from the general population indicate that lutein consumption by adults is 0.8–1.1 mg/day, 0.5–4.0 mg/day, 0.6–1.1 mg/day, 1.51 mg/day, and 2.9 mg/day in United States, Europe, Brazil, Japan and China respectively [51,52,53,54,55,56]. In 2017 more than one third of the European population did not eat fruit and vegetables on a daily basis [57]. The European Food Safety Agency (EFSA) database on fruit and vegetables (F&V) intake in children 36 months to 9 years of age indicates that in all the member states the consumption is substantially below the WHO recommendations of eating a minimum of 400 g of F&V per day [58]. Data from the NHANES 2003–2004 showed that the intake of lutein + zeaxanthin was less than 0.6 mg/day in children and adolescents (1–18 years of age) and less that 2 mg/day in women of childbearing age [52]. More recent data from pregnant women between 19–43 years old showed that maternal lutein and zeaxanthin intakes averaged 2.48 mg/day [59].

In a cross-sectional study conducted in Italian women aged 20–25 years the mean intake of lutein and zeaxanthin were approximately 1 mg/day [60]. Slightly higher intakes (1.2 mg/day) were observed in middle-class and well-educated women aged 24–42 years at day 3 postpartum [61] (see Table 1).

Dietary Carotenoids are absorbed in the small intestine. As lipophilic molecule, their absorption follows the same processes of other fat-soluble dietary compounds such as dietary lipids or vitamin E. The concomitant ingestion of some fat is necessary for optimal absorption. Lutein and zeaxanthin absorption varies considerably among individuals and is influenced by external and host factors that may interfere with the different steps involved in its absorption: source (free or esterified), amount and presence of other carotenoids, duration of intake, amount of fat in the diet (substantially higher amount of fat is required for the absorption of lutein and zeaxanthin esters compared to the most common dietary free forms), concomitant ingestion of fibers, nutritional status, genetic factors and the age of the individual [62]. Lutein and zeaxanthin in the free forms are directly absorbed by the body. The esterified forms must be hydrolyzed before absorption and this additional step seems to be not 100% efficient requiring the concomitant ingestion of atypically higher fat [38,63,64,65]. In brief, after release from the food matrix, the molecules are incorporated into mixed micelles formed from dietary fats and bile acids. Lutein and zeaxanthin are then absorbed by the mucosa of the small intestine via passive or facilitated diffusion, incorporated into chylomicrons, and transported via the lymphatic system to the liver. Finally, in the hepatocytes, lutein is incorporated into lipoproteins (maily low and high density lipoproteins (LDL and HDL) and transported to target tissues. Lutein is able to cross the blood-retina barrier, blood-brain barrier and the placenta [11]. The distribution of carotenoids in human organs shows specificity [30]. Xanthophylls form a bit less than 20% of the total carotenoids in the human diet. Already in the blood plasma, the amount of xanthophylls increases to about 40% [35]. This preferential selection of lutein and zeaxanthin, and particularly lutein, is further enhanced at the level of the developing neural tissues as they are preferentially accumulated from circulation into the brain [66,67] and retina [68,69].

Specific accumulation of these xanthophylls also occur in breast milk [70]. The differences in lutein and zeaxanthin dietary intake and the potential nutritional gaps discussed above reflect the carotenoids concentration in breast milk which can vary up to 10 times within the same population [71,72].

### 2.3. Mechanism of Action

Diets with adequate intakes of carotenoid-rich foods have been linked to possible protection against non-communicable diseases [73,74,75]. Lutein and zeaxanthin research has focused on the beneficial effects for eye and brain aging [39,40,46,48,66,76,77] and more recently researchers have started to explore their effect for vision and cognition in young adults and early life [44,45,49,78,79,80,81,82,83].

The molecular structure of lutein and zeaxanthin described previously determines the natural function and the mode of action in living organisms. The molecule size, shape and the presence of the hydroxyl groups are important for the antioxidant effect, interaction and orientation in cellular membrane [32]. Lutein and zeaxanthin’s inherent structure allows for integration into cellular membranes in a way non-polar carotenoid such as β-carotene or lycopene cannot. Specifically, Sujak et al. showed that lutein and zeaxanthin have different orientations within the membrane: lutein can be oriented either perpendicularly or parallel in the bipolar cell membrane, while zeaxanthin has only the perpendicular orientation in the membrane [84]. The Authors suggested that lutein’s ability to have two orientations in the bipolar membrane may make it ideally suited to provide protective abilities.

Carotenoids are among the most effective quenchers of the singlet oxygen, can also quench peroxyl and tocopheryl radicals, and inhibit lipid peroxidation; the presence of electrons localized over the polyene chain allows for neutralization of free radicals [32,85]. Associated with their location within the cell membrane lutein and zeaxanthin may protect the cell from oxidative damage via its AO and light absorption properties. This may be particularly important for the eye and brain tissues (especially in early life and in prematurity) which are more susceptible to oxidative damage due to the abundance of polyunsaturated fatty acids such a docosahexaenoic acid (DHA) in cellular membranes, high oxygen consumption, high metabolic and functional properties and (in the case of the eye) direct exposure to light.

Preclinical studies have shown that lutein can protect the neural tissues from chemical-induced hypoxia and cell apoptosis, hydrogen peroxide and streptozotocin-induced OS, ischemia-reperfusion injury [86,87,88]. OS and inflammation are closely related pathophysiological events that are tightly linked with one another.

Several reactive oxygen/nitrogen species can initiate intracellular signaling cascades that enhance proinflammatory gene expression. Conversely, inflammatory cells liberate ROS at the site of inflammation leading to exaggerated OS [89]. Lutein has been also shown to modulate the inflammatory response in retina, brain, skin and immune cells [90,91,92,93]. Furthermore, by preventing lipid peroxidation lutein can protect and preserve DHA and make it available for conversion to anti-inflammatory molecules [94].

The specific ability of lutein and zeaxanthin to absorb high-energy short wavelengths of visible light (blue light) have an impact on vision beyond the protection of photoreceptor from blue-light induced production of ROS. Blue light absorption can positively influence visual function by attenuating chromatic aberration, light scatter in the eye associated with disability glare, blue haze and improve contrast sensitivity [25,33,44,95,96].

Finally, carotenoids, influence gap junctional communication [97,98]. This characteristic can be an important complement to the mechanism of action of lutein and zeaxanthin. Gap junctions are transport channels between cells, particularly neuronal cells like those found in the human eye. The channels are important because they permit electrical and metabolic coupling of cells joined maintaining homeostasis within coupled cells in times of stress such as might occur in the maturing infant eye and brain [99].

### 2.4. Safety

Lutein is a very safe molecule with an extensive history of consumption from the diet at varying levels of intake. In several toxicity studies, including developmental toxicity and dermal irritation, no adverse effects were documented in animals, including monkeys or humans [100,101,102,103].

There have been hundreds of peer-reviewed published human studies involving lutein supplementation alone or in combination with zeaxanthin, other carotenoids, and AO. The highest lutein dosage administered (40 mg/day for nine weeks) and the longest duration of supplementation (10 years supplementation with 10 mg lutein + 2 mg zeaxanthin) have been assessed in clinical trial on eye-related diseases [39,42,104].

All these data were considered by different authorities around the world. The initial safety review conducted by the Joint Food and Agricultural Organization (FAO)/World Health Organization (WHO) Expert Committee on Food Additives (JECFA) established an Acceptable Daily Intake (ADI) for lutein and zeaxanthin from *tagetes erecta* of 2 mg/kg body weight/day (140 mg/day for a 70 kg person) and has been recently updated to reflect the established safety of lutein and zeaxanthin providing a “non-specified” ADI [100]. In Europe, lutein and zeaxanthin are considered traditional ingredients used in food, beverages, and food supplements. The European Food Safety Agency (EFSA) established an ADI of 1 mg lutein /kg body weight for adults and children. Furthermore, it was determined to be Generally Recognized as Safe (GRAS) in the USA for inclusion in food products and infant formulas and additionally, EFSA has established the safety and bioavailability of lutein in infant formulas [101,103].

## 3. Lutein in Pregnancy and Breastfeeding

Research conducted in diverse populations around the world have shown that lutein and zeaxanthin are present in maternal plasma throughout the course of pregnancy, persist during the postpartum period and are transferred to the developing fetus via the cord blood [70,105,106,107,108]. Recently, Thoene et al. [59] indicates that lutein and zeaxanthin combined are the most prevalent carotenoids in placenta (49.1% of the tested carotenoids) and umbilical cord blood (37.0%), although they are less prevalent in maternal serum (18.6%) or diet (19.4%), with a rate of transfer of 16.0%, the highest of all carotenoids. The presence of lutein and zeaxanthin in placenta can improve its strength, stability, permeability and decrease lipid peroxidation offering protection and nourishment of the developing fetus.

Oostenburg et al. [109] by looking at the concentration of several antioxidants and carotenoids during all trimester of pregnancy found that among the carotenoids analyzed only lutein level increased by 41% from the first to the third trimester and remained elevated after delivery. Lutein and zeaxanthin concentration in cord blood were found to be correlated with maternal plasma concentration although their levels were much lower [109,110].

Nature continues to provide lutein and zeaxanthin to the developing baby after birth. The colostrum, the first mother’s milk, is rich in carotenoids which provide its characteristic yellow color. Lutein and zeaxanthin have been found in breast milk throughout the entire nursing period and like other carotenoids their concentration is lower in mature milk than in colostrum and early milk [61,111,112]. As for plasma levels, their concentration in breast milk are significant correlated with dietary intakes and correlated to each other [61,70].

In 1990 Patton and colleagues analyzed the colostrum of 11 women within 6 days after delivery and discovered that colostrum contains lutein, zeaxanthin, β-carotene, lycopene, and β-cryptoxanthin [113]. Levels of these carotenoids in milk were later quantified by Khachik et al. in 1997 [29] who analyzed serum and breast milk carotenoid concentration of three women one month postpartum. Of the carotenoids found, lutein had the third highest concentration in the serum while it was the most abundant carotenoid in breast milk together with zeaxanthin. Lutein was 2–3 times more concentrated than ß-carotene in breast milk, whereas the concentration was approximately the same in maternal plasma. This finding led to the hypothesis that lutein may be actively secreted into milk. Preferred deposition of lutein and zeaxanthin in breast milk has been additionally confirmed by Sherry et al. [70]. The study was conducted to determine the impact of lutein supplementation in the breast milk and plasma of lactating women and in the plasma of breast-fed infants 2–3 months postpartum. Baseline observations on maternal samples indicated that while total lutein + zeaxanthin ranked third in maternal plasma (following total lycopene and β-carotene) they were the most abundant carotenoid in breast milk. Interestingly, the carotenoid distribution in the plasma of infant at baseline closely matched that of breast milk. Recent observations from a Chinese cohort provided additional indication that lutein is the predominant carotenoid in transitional and mature milk [114].

The last trimester of pregnancy and lactation are two critical periods for retina and brain development. The increase in concentration of lutein and zeaxanthin in these periods coupled with their preferential deposition in both organs suggests an important biological role.

Exclusive breastfeeding is recommended for feeding infants at least during the first six months of life. When this is not possible for mothers infant formula can be the primary source of nutrition for the baby. The presence of lutein in infant formula is limited to some regions of the world. In Europe, lutein is not added to artificial milk and this is a key differentiating factor between breastmilk and infant formula composition which may result in low plasma lutein levels in formula-fed infants. Bettler et al. [115] showed that breastfed infants had approximately 6 times more the mean serum lutein concentration of infants fed unfortified milk formula. In the same study, the addition of lutein to artificial milk (in doses of 25 mcg to 200 mcg per L) increased serum lutein concentration in a dose-dependent way. However, in order to achieve the same serum concentration observed in breast fed infants approximately 4 times more lutein is needs in the infant formula. Six weeks of maternal supplementation with 6 mg or 12 mg lutein during breastfeeding resulted in: (i) increased total lutein and zeaxanthin in maternal plasma and breast milk, and (ii) increase of these carotenoids in infant plasma in a dose-dependent manner [70]. Twelve weeks supplementation of breastfeeding mothers with a multiple micronutrient supplement containing very low amounts of lutein (250 ug/day) vs. placebo was found to to prevent the decline in maternal plasma of lutein concentrations. Furthermore, while a decrease in lutein levels in breast milk during lactation was observed in mothers supplemented with placebo, no decrease was observed in the group receiving low dose lutein [116].

Emerging data from research in non-human primates indicates that lutein supplementation lead to increased deposition of this carotenoid in the developing eye and brain tissues [117,118] and mother-infant human observational studies suggest a role of lutein in visual and cognitive function [80,81].

A prospective randomized controlled trial is currently being conducted to evaluate the effects of supplementation with 10 mg lutein + 2 mg zeaxanthin administered for 6 to 8 months during pregnancy (from the first trimester of pregnancy to 2 weeks postpartum). The study aims to assess wheter supplementation will counteract maternal carotenoid depletion during pregnancy and will improve biomarkers of carotenoid status (serum, MP and skin carotenoids) and ocular health of both mothers and infants [119].

## 4. Lutein in Eye and Brain Development and Function

### 4.1. Search Method

A narrative Literature review was conducted on PubMed through 21 June 2021 to identify human studies evaluating the effect of lutein and zeaxanthin on visual and/or cognitive function in a healthy pediatric population.

The search terms were ((lutein OR zeaxanthin OR MPOD) AND (eye OR brain OR vision OR cognition) AND (children OR infant OR adolescent OR child)). Inclusion criteria were observational studies or intervention trials conducted in humans and reporting on the effect of dietary, plasma or retinal lutein and zeaxanthin on visual or cognitive outcomes in healthy term infants, children or adolescents.

Among the 152 studies retrieved, 9 publications met the inclusion criteria. Two articles, discussed in Section 4.2.3, assessed visual outcomes [80,120] and 7 papers, reported in Section 4.3.3 and Table 2, presented findings related to cognitive performance [81,82,83,121,122,123,124]. Three additional publications explored the effect of lutein supplementation in premature infant with or without retinopathy of prematurity. These papers weres considered important and were presented in Section 4.2.4.

Of the additional articles retrieved, the papers presented data on (1) the effect of lutein and zeaxanthin supplementation in non-human infant primates, (2) their distribution in the eye and brain, (3) techniques for MPOD measurement in the pediatric population and (4) intervention studies exploring the effect of lutein and zeaxanthin supplementationm and cognition conducted in a healthy adult population, were also considered important for the review and were discussed. Reviews providing repetitive information were generally not considered. In addition, pre-clinical studies (other than non-human primates), articles reporting findings from studies conducted in a disease population, or not reporting cognitive or visual outcomes related to lutein, zeaxanthin or MPOD were not presented. Further publications were gathered from a manual search of the reference list of articles and reviews.

### 4.2. Lutein, Eye Development and Visual Function

#### 4.2.1. Eye Deposition

The human eye accumulates the dietary carotenoids lutein and zeaxanthin. The maximum concentration of these pigments (about 70% of their total content in the eye and more than a thousand times their concentration in serum) is observed in the *macula lutea* [125]. Among the dietary carotenoids, only lutein and zeaxanthin are selectively deposited in this pigmented area of the retina responsible for high acuity color vision. A third non-dietary zeaxanthin isomer meso-zeaxanthin [(3R,3′S)-β, β-carotene-3,3′-diol] which originates from lutein conversion in the retina contributes to the composition of the macular pigment (MP) [126]. MP reaches the highest concentration (per mm^2^ of tissue) at the center of the fovea decreasing rapidly with distance from the epicenter [68,126]. Specific xanthophylls binding proteins—Steroidogenic acute regulatory domain protein 3 (StARD3) for lutein and Glutathione S-transferase P1 (GSTP1) for zeaxanthin and meso-zeaxanthin—mediate the selective uptake, distribution, and stabilization of the macular carotenoids in this tissue [125,126,127]. BCO1 (β-carotene-15,15-monooxygenase) and CD36 (cluster of differentiation 36) genes were found to be also implicated in plasma and retina concentrations of lutein in adults and more recently children [128,129].

The term macular pigment optical density (MPOD) refers to a measurement of the attenuation of blue light by macular pigment (expressed in density units, du) and provides an indication of the amount of lutein and zeaxanthin isomers in the macula. Typical MPOD levels vary between 0 and 1 du [33]. The concentration of macular carotenoids can be measured in the macula of donor eyes with high performance liquid chromatography (HPLC) while in living eyes MPOD can be assessed non-invasively using subjective psychophysical techniques, such as heterochromatic flicker photometry, and objective optical methods, such as fundus autofluorescence and reflectometry [33]. In children up to the age of 7 years, due to the difficulties in performing psychophysical task reliably, objective measurements are preferred, and blue light reflectometry offers the opportunity to image the MP of infants and children [34].

Lutein and zeaxanthin have been detected in the eye as early as the second trimester of gestation. Yakovleva et al. [125] found these xanthophylls the vitreous body of the developing human eye from week 15 until week 28 WG. Lutein and zeaxanthin disappear from the vitreous in the last trimester of pregnancy (lutein and zeaxanthin are not present in the vitreous in post-natal age or in adults) in association with the gradual accumulation in the retina and the lens and the correspondent tissue development and differentiation. Lutein and zeaxanthin concentration as well as distribution in the foveal area has been shown to change as the foveal area matures. MPOD has been first detected at postmenstrual age of 33 WG at the time the foveal pit starts to form and continues to increase as the fovea matures up to the age of 3–4 years when it is essentially equivalent to the amount found in the adult eye [5,69].

Lutein is the dominant carotenoid in the infant retina during foveal maturation process with a ratio of lutein to zeaxanthin isomers close to 1.6 at birth, and tends to approaches adults’ distribution of 0.5 after the age of 3 [69,130]. The changes in the lutein/zeaxanthin ratio in the macula appear to be closely related to steps in the anatomical and functional development providing additional support for a biological and functional role [69,126].

#### 4.2.2. Pre-Clinical Research in Non-Human Primates

The importance of lutein and zeaxanthin to the development of the visual system and the potential synergism with other key nutrients such as DHA, were explored in research conducted in non-human primates raised on a xanthophyll-free diet otherwise containing the adequate amounts of calories, vitamins and minerals, from pre-natal development and after birth. Compared with animal raised on a standard diet containing lutein and zeaxanthin, xanthophyl-free animals were found to present: (1) undetectable levels of lutein and zeaxanthin in serum, (2) no yellow macular pigmentation, (3) distinct changes in the RPE cell profile (foveal dip) and density (increased cell density), (4) increase in macular hyperfluorescence and mottling of the RPE although in absence of major visual disturbances, and (5) were more vulnerable to damage in the foveal region induced by acute blue-light exposure [131,132,133,134]. Supplementation with purified lutein or zeaxanthin resulted in increased serum levels of these xanthophylls, accumulation of MP (constituted by lutein and meso-zeaxanthin in the animals fed purified lutein and only zeaxanthin in the animals fed purified zeaxanthin), attenuation of the structural changes in RPE, and decreased foveal sensitivity to blue-light exposure [132,133,134] (see Table 2).

Additional studies conducted in infant monkeys breast fed or fed a formula containing high or low amounts of lutein and zeaxanthin indicate that the bioaccumulation of lutein and zeaxanthin in the retina is higher when provided in breast milk [117,118].

#### 4.2.3. Lutein, Oxidative Stress and Visual Function in Humans

The AO and anti-inflammatory characteristics of lutein and zeaxanthin coupled with their blue-light absorption properties can benefit a tissue particularly susceptible to OS early in life such as the retina. These effects can potentially attenuate the rate of increase in lipofuscin formation in the RPE, found to be greater in the first decade of post-natal and later in life [7,22,135].

A retrospective observational study explored the effect of supplementation with 10 mg lutein + 2 mg zeaxanthin or no supplementation in 24 pregnant women with gestational diabetes. Lutein and zeaxanthin intake was associated with a non-statistical significant decrease in maternal plasma total hydroperoxides (TH) and a significant decrease in plasma TH in the newborns at 2 h of life [136].

Henriksen et al. showed that correlations exist between mother and infant serum lutein and zeaxanthin and between maternal and infant serum zeaxanthin and MPOD [137].

The relationship between maternal lutein and zeaxanthin intakes and the off-spring eye maturation has been recently explored in humans. In the GUSTO study (Growing Up in Singapore Towards healthy Outcomes), an observational study was conducted to assess the relationship between maternal lutein and zeaxanthin plasma concentrations during pregnancy and visual acuity (VA) in the offspring at 3 year of age in 471 mater-child pair. Authors found that higher maternal lutein and zeaxanthin plasma concentrations during pregnancy were associated with lower likelihood of poor distance-VA in children. They concluded that maternal lutein and zeaxanthin status during pregnancy may influence a baby’s early visual development [80].

In an observational study conducted in 94 healthy Chinese children 6 to 12 years old, Zheng et al. explored the relationship between MPOD, refractive status and foveal thickness. No correlation between MPOD spherical equivalent refraction or foveal thickness was observed in this population. However in children low-to-moderate myopia MPOD was inversely related to minimum foveal thickness and positively related to central foveal thickness [120].

Neuringer et al. showed that serum lutein and zeaxanthin levels and MPOD were higher in infants fed with breast milk than those with infant formula [138]. Other Authors reported that early exposure (via breast milk) to lutein and zeaxanthin appears to influence MPOD later in life [139].

Research over the past decades has demonstrated that a thicker MP, or higher MPOD, is associated with better visual performance. Intervention studies in a young adult population have shown that lutein and zeaxanthin supplementation increase MPOD and benefit visual function in terms of glare sensitivity, contrast sensitivity, photostress recovery, visual fatigue as well as influencing visual processing at cortical levels [43,78,79,140,141,142,143].

The presence of lutein and zeaxanthin in the macula during the early stage of life is important considering that children are particularly susceptible to the potential damaging effects of excessive light exposure (solar light or artificial LED light) due to the immature eye development and the complete transparency to light of the ocular lens leading to increased risk for OS.

All these observations support the recommendations for an increased supply of lutein and zeaxanthin during development and early life. Studies conducted in newborns have shown that the oral administration of lutein (either through lutein fortified infant formula or separately added to the infant milk) was well tolerated, resulted in increase in plasma lutein levels and provided benefits in terms of reduction of oxidative stress [144,145,146,147,148,149,150]. In the study by Perrone et al. [149] conducted in 150 healthy term infants, the administration of a lutein supplement at 6 and 36 h after birth resulted in increased biological AO potential and lower levels of OS assessed at 48 h of life compared to placebo. Results confirmed a previous observation from a pilot study conducted on 20 healthy term infants using a similar study design [150].

#### 4.2.4. Lutein in Premature Infants

In 2014 the estimated global preterm (<37 weeks of gestation) birth rate from 184 countries was 10.6% (range 8.7–11.9%). Regional estimates indicate a 6.3–13.3% premature births in Europe and 9.5–13.2% in North America [16]. Preterm infants (PI) are generally at higher risk of visual impairment due to exposure in the delivery room or in NICU to potentially damaging concentrations of oxygen and high light intensity which may favor OS and free radical production [11,151,152,153]. OS is the main consequence of retinal ischemia and may have a role in pathologic angiogenesis of the retina and thus in the pathogenesis of retinopathy of prematurity (ROP) [151], the second cause of childhood blindness after impaired cortical vision [152]. OS associated with the unbalance between the production of free radicals and the detoxifying capability of the AO system is also involved in the damage observed in other areas of the central nervous system (CNS) [151,152]. One of the goals in the management of PI is to reduce the production of free radicals and promote concomitantly the development of the AO system [153] and its role in the management of ROP [151] Because of lutein AO, anti-inflammatory, blue light absorption properties and the well know role in eye health, its beneficial role in this condition has been hypothesized.

Preterm delivery exposes infants to a disadvantage in terms of lutein and zeaxanthin supply because they miss the period of maximal placental delivery of these antioxidant nutrients. Initial assessments of carotenoids status conducted in PI found very low or almost undetectable macular pigment and low serum and skin carotenoids levels [154]. Sasano et al. [5] by exploring the MP levels and changes over time in 40 premature infants detected MP in 39 out of 40 infants (MPOD values ranged from 0 to 0.18). MPOD value greater than 0 was first detectable at 33 WG; MPOD levels increased linearly as a function of infant growth and retina maturation (starting most probably around 26 WG when the foveal pit starts to form) and the growth patterns for both eyes were similar.

Lutein supplementation in PI has been shown to increase serum lutein levels [144,148] and decrease plasma C-reactive protein [144]. Plasma lutein concentrations were found to correlate with total AOs status and saturated response amplitude in rod photoreceptors [144,155]. Furthermore, in PI with no ROP lutein supplementation resulted in greater photoreceptor sensitivity [144]. Preliminary indications for a lower threshold for ROP, NEC and BPD were observed in infants receiving lutein and zeaxanthin supplementation [156,157]. However, despite of these encouraging results none of the clinical trials conducted to date showed any statistical significant reduction in ROP incidence nor the risk of BPD, sepsis, NEC and mortality [158].

### 4.3. Lutein, Brain Develeopment and Cognitive Function

#### 4.3.1. Brain Deposition

Lutein and zeaxanthin cross the blood-brain barrier and are deposited in the brain. In 2004, Craft et al. identified and measured a broad range of AOs in the adult brain and showed that the xanthophylls lutein, zeaxanthin and cryptoxanthin accounted for 66–77% of the carotenoids in the brain regions studied. Since these carotenoids are less predominant in human diet or blood, Craft first suggested a preferential accumulation of oxygenated carotenoids in the brain [159]. More recently, it has been shown that: (1) xanthophylls make up 72% of total carotenoids in the adults brain, (2) lutein alone accounts for over one third (34%), significantly greater than other carotenoids [66], and (3) lutein is the predominant carotenoid in the infant brain representing 59% of the carotenoids and reaching 74% of the total carotenoids when combined with zeaxanthin [67].

The analysis conducted on brain tissues (hippocampus, prefrontal, frontal, auditory, and occipital cortices) from 30 infants who died during the first 18 months of life [67] found that lutein, zeaxanthin, cryptoxanthin and β-carotene were the major carotenoids. Lutein was significantly more concentrated than all the other carotenoids in all the brain regions analyzed and its concentration was higher than that of all the other carotenoids combined. Moreover, lower concentrations of lutein, zeaxanthin, and cryptoxanthin in most brain region analyzed have been found in PI than in term infants. These findings, were consistent with the limited in utero accretion of lutein and zeaxanthin (higher in the last trimester of pregnancy) associated with prematurity.

An exploratory metabolomics analysis was conducted on postmortem human infant brain tissues to elucidate potential mechanisms through which lutein may influence neurodevelopment. Lieblein-Boff et al. [160] observed that: (i) lutein and its isomer zeaxanthin were the only carotenoids present in all infant brain regions studied, (ii) lutein concentrations in frontal cortex, hippocampus, and occipital cortex are correlated with a number of metabolites in a brain region specific manner, (iii) lutein correlated with lipid pathway metabolites, energy pathway metabolites, brain osmolytes, amino acid neurotransmitters, and the AO homocarnosine in brain-specific regions, and (iv) lutein is concentrated in neural tissues important for learning and memory. Results indicate that lutein may be related to brain volume regulation during growth and development, myelination, neurotransmission, development or remodeling of neurons and AO neuroprotection.

Recently Tanprasertsuk et al. [161] reported, in infants, the highly significant cross-sectional relationship between brain concentrations of lutein and the levels of the binding protein StARD3 (previously identified as the specific binding protein for lutein in retinal tissues). Authors suggested a possible mechanism for the selective accumulation of lutein in the brain and further supportive role for lutein in early neural development.

The eye-brain link in terms of lutein accumulation is strengthen further by observational human studies. Results showed associations between lutein concentration in the retinal region and its concentrations in the occipital cortex, the primary visual processing area of the brain. Moreover, correlations between serum and brain levels of the carotenoids lutein, zeaxanthin cryptoxanthin and ß-carotene have been also found [162]. Notably, in primates, macular–brain associations were found for lutein and zeaxanthin and the cerebellum, lutein and pons, as well as for zeaxanthin and the frontal cortex [163]. 

#### 4.3.2. Pre-Clinical Research in Non-Human Primates

In infant rhesus monkeys breast fed or fed a formula containing added lutein (in a concentration comparable to breast milk) or not enriched, lutein was differentially distributed across all the brain region evaluated (prefrontal cortex, occipital cortex, superior temporal cortex, striatum, cerebellum, motor cortex, gray matter, white matter, and hippocampus). The highest amount of lutein was found in the occipital cortex regardless of the diet, suggesting lutein’s role in visual processing in early life. Breast feeding resulted in higher brain deposition of lutein compared to formula feeding indicating that maternal milk is the preferred dietary vehicle for the delivery of lutein. This observations reinforce the importance of ensuring an adequate maternal lutein intake. In infant monkeys fed with the lutein-enriched formula brain lutein levels were significantly higher than unsupplemented animals [117,118].

Lutein was also found to be the predominant carotenoid in subcellular membranes in cerebellum, hippocampus striatum and prefrontal cortex tissues of adult rhesus monkey fed stock diet or lutein/zeaxanthin supplemented diets. Lutein was the only carotenoid detected in all the membranes analyzed (nuclear, myelin, mitochondrial and neuronal plasma membranes). Accumulation was especially evident in the mitochondrial membranes, particularly in the prefrontal cortex where its levels were inversely associated with DHA oxidation products suggesting an important antioxidant role associated with DHA protection in the brain [164].

#### 4.3.3. Lutein Status and Cognitive Function in Humans

MPOD, considered a stable measure of lutein and zeaxanthin in neural tissues, has been associated with better global cognition, verbal learning and fluency, and processing and perceptual speed in healthy young and old people [78,79,94,165,166,167,168,169]. Lutein and zeaxanthin plasma or retinal levels have been associated with enhanced neural efficiency, brain activation, white matter integrity [170,171,172]. Interventional studies also provide support that supplementation with lutein and/or zeaxanthin may enhance cognitive function and help maintain cognitive health. A total of 13 randomized, double-blind, controlled interventional studies have been conducted to date investigating the role of lutein and zeaxanthin intake (supplementation or dietary intervention) on cognitive functions in healthy young and more mature adults [46,47,49,77,78,79,171,173,174,175,176,177,178]. Of them, four were conducted in a young student population (adults) [49,78,79,174]. These studies have shown the effect of supplementation in improving critical flicker fusion thresholds and missed coincidence anticipation time—two measures of neural processing speed and visual processing speed—visual memory, complex and sustained attention, reasoning abilities and other parameters of cognitive performance. A systematic review of randomized controlled trials [179] and a meta-analysis [180] suggest that daily supplementation with lutein (mainly administered in a 10 mg daily dose as food supplement) and its isomers can improve cognitive functions.

The encouraging findings of a positive impact of lutein and zeaxanthin on brain function in the adult population coupled with their preferential deposition in the infant brain has suggested the putative role early in life and has prompted research to elucidate the implications of lutein and zeaxanthin status and children’s cognitive function.

A cross-sectional study assessed the distribution of lutein and activin A, a well-established neurobiomarker of CNS development and damage, in arterial cord blood of healthy PI and term infants. A significant and positive correlation between the two groups was observed in male and female suggesting a neurotrophic role of lutein [108]. High levels of activin A and lutein were detected in early weeks of the third trimester of gestation when CNS development starts to be at its highest level in terms of brain volume, weight and structure and progressively decrease with the end of pregnancy.

Higher gestational lutein intakes were associated with better child behavior regulation in a prospective study on 1580 mother-child pairs [81]. High lutein and choline concentrations in maternal breast milk have been found to be associated to better recognition memory in 6-month old infant [82]. The Authors suggest that this cognitive advantage observed in infants ingesting more lutein could be associated with a more rapid development of the visual system. Furthermore, similar to adults, high MPOD levels were associated with different measures of cognition including global intelligence and executive processing, memory and academic achievement in school children [83,122,123,124]. An overview of the major findings from the studies exploring the relationship between lutein and zeaxanthin status and cognitive performance are reported in Table 3.

Intervention trials to confirm the cause-effect relationship between lutein intake and cognitive outcomes in the pediatric population are awaited.

## 5. Conclusions

Lutein and zeaxanthin are unable to be synthesized in humans, so blood and tissue levels rely solely on dietary intake. However, these carotenoids are not considered essential so, there is no dietary reference intake established for lutein to date. There is growing support for setting intake recommendations for non-essential dietary bioactives, such as lutein, which promote optimal health and/or prevent occurrence of chronic diseases [181,182]. Studies on eye and brain health and function conducted in a young adult population show the benefits of 10 mg of lutein and 2 mg of zeaxanthin daily. Maternal supplementation with the same doses during the third trimester of pregnancy resulted in reduced OS in the newborn that is at high risk since AO defense is deficient in newborns. Furthermore, maternal lutein supplementation during breastfeeding results in increased serum lutein and zeaxanthin level in the mother and in the breastfed infant. Breastfeeding is recommended for feeding infant at least during the first six months of life and appears to be far superior for lutein absorption and bioavailability than infant formulas. Furthermore in Europe, milk formulas are not enriched with lutein, a key difference to breastmilk composition, which may result in low plasma lutein levels in exclusively formula-fed infants.

The mother should have an adequate intake of lutein and zeaxanthin to supply her own needs and that of her baby. Higher maternal lutein and zeaxanthin intake or serum level have been associated with potential beneficial effects for visual acuity and cognitive behavior during childhood.

Children with higher lutein and zeaxanthin status reflected by higher MPOD were found to have better cognitive performance then their low MPOD peers. Data from food intake studies indicates that women of childbearing potential, breast feeding women and children do not ingest the amount of fruit and vegetable required to ensure an adequate intake of lutein and zeaxanthin. Recommendations to add dark green leafy vegetables to the diet of pregnant and breastfeeding women and their child or complement the diet with prenatal and pediatric supplement can help bridge the gap.

## Figures and Tables

**Table 1:** Estimates of L and Z intakes by individuals consuming the number of serving of F&V recommended by dietary guidelines versus actual levels of intakes reported in observational studies (mg/day).

Population	Age	L + Z Estimated Intake Based on Recommended 3–5 Servings F&V/day	Average Daily L + Z Intakes
General population, USA [50]	2 years and older	Mean 3.83 90th percentile 7.29	-
General population, USA [52]	1–18 years	-	Mean < 0.6
Females of childbearing potential, USA [52]	19–50 years	-	Mean < 2
Pregnant women, USA [59]	19–43 years	-	2.48
Pregnant women, Italy [60]	20–25 years	-	1
Breastfeeding women, Italy [61]	24–42 years	-	1.2

Abbreviations: L, Lutein; Z, zeaxanthin; F&V, fruit and vegetables.

**Table 2:** Key finding from studies conducted in non-human primate raised on a diet devoted of xanthophylls (xanthophyll-free animals) supplemented with lutein and zeaxanthin.

Outcome	Xanthophyll-Free Animals	L/Z, L or Z Supplemented Animals
Serum levels	Undetectable levels of L and Z	Increase levels of L and/or Z
Macular pigment	No yellow macular pigmentation	Accumulation of macular pigment
Retina	Distinct changes in the RPE cell profile (foveal dip) and density (increased cell density). Increase in macular hyperfluorescence and mottling of the RPE although in absence of major visual disturbances. Prominent presence of drusen-like bodies at the level of the pigment epithelium.	Attenuation of the structural changes in RPE cell profile (central foveal peak), presence of asymmetry in the RPE profile suggesting that L and Z could stimulate cell migration.
Blue Light sensitivity	Increased vulnerability to acute blue-light induced damage in the foveal region	Decreased foveal vulnerability to acute blue-light exposure

Abbreviations: L, Lutein; Z, zeaxanthin; RPE, Retinal Pigment Epithelium.

**Table 3:** Observational studies addressing the relationship between lutein and zeaxanthin status and cognitive function in a pediatric population.

Author	Year	Age	*n*	Key Findings
Mahmassani [81]	2021	Pregnancy I trimester (median 9.9 WG) Pregnancy II trimester (median 27.9 WG) Infancy (5.2–10.0 months) Early-Childhood (2.8–6.2 years) Mid-Childhood (6.6–10.9 years)	1580 mother-child pairs	Greater maternal L/Z intakes in the I-II trimester were associated with better verbal intelligence (main analysis) and better behavior regulation ability (secondary analyses) in mid-childhood. Higher maternal I trimester intake of L/Z-rich foods was associated with better social-emotional development and behavioral regulation ability in this same age group. No benefits of greater maternal L/Z intakes were observed in infancy and early childhood
Saint [124]	2018	7–13 years	51	Link between higher carotenoid status and improved cognitive functioning. MPOD was significantly correlated to global Intelligence (Brief Intellectual Ability) and executive processes composite scores. Exploratory analysis also showed positive associations with spatial relations subtest.
Barnett [83]	2018	8–9 years old	56	MPOD is positively related to academic achievement, mathematics, and written language composite standard scores in school children.
Walk [123]	2017	8–10 years	49	MPOD is correlated (*p* < 0.05) with cognitive control performance. Children with higher MPOD present higher accuracy in performing tasks which require cognitive control processing (modified flanker task) and require the allocation of less attentional resources to perform the task (smaller P3 amplitudes in the EEG recordings).
Hassevoort [122]	2017	7–10 years	40	MPOD is positively associated with a spatial reconstruction task designed to assess relational memory performance, a hippocampal-dependent function, even after accounting for IQ and aerobic fitness.
Cheatham [82]	2015	6 month-old	55	High L & High Choline in maternal breast milk are associated with better infant recognition memory (difference in latency to peak amplitude scores at frontal and central areas in EEG recordings (*p* < 0.05 and *p* < 0.001; respectively)
Mulder [121]	2014	5.6–5.9 years	160	L intake and L serum levels showed no association with child cognitive tests.

Abbreviations: L, Lutein; Z, zeaxanthin; MPOD, Macular Pigment Optical Density; EEG, electroencephalography; IQ, Intelligence Quotient.
